# Diversity Analysis of the Sugar Beet Pathogens ‘*Candidatus* Arsenophonus phytopathogenicus’ and ‘*Ca*. Phytoplasma solani’

**DOI:** 10.3390/plants15111618

**Published:** 2026-05-25

**Authors:** Rafael Toth, Theresa Kaufmann, Matthias Schulten, Sonja Dunemann, Bruno Huettel, Mark Varrelmann, Michael Kube

**Affiliations:** 1 Department of Integrative Infection Biology Crops-Livestock, University of Hohenheim, 70599 Stuttgart, Germany; theresa.kaufmann@uni-hohenheim.de (T.K.); matthias.schulten@uni-hohenheim.de (M.S.); 2 Max Planck-Genome-Center Cologne, Max Planck Institute for Plant Breeding Research, 50829 Cologne, Germany; sdunemann@mpipz.mpg.de (S.D.); huettel@mpipz.mpg.de (B.H.); 3 Department of Phytopathology, Institute of Sugar Beet Research (IfZ), 37079 Göttingen, Germany; varrelmann@ifz-goettingen.de

**Keywords:** SBR, *Beta vulgaris*, *P. leporinus*, SMRT, long-read sequencing, diversity, marker

## Abstract

Sugar beet cultivation in Europe is threatened by two vector-borne diseases: syndrome “basses richesses”, caused by the phloem-limited pathogen ‘*Candidatus* Arsenophonus phytopathogenicus’, and phytoplasmoses associated with ‘*Ca*. Phytoplasma solani’ subgroup 16SrXII-A and the related subgroup 16SrXII-P. Infections lead to reduced sugar yield, biomass and growth abnormalities. In Germany, *Pentastiridius leporinus* represents the main vector. Despite their importance, genetic diversity remains poorly understood. During a two-year survey, barcoded amplicons were generated from infected sugar beet samples from Germany and neighbouring countries using the phytoplasma markers 16S rRNA-ITS-23S rRNA, *tuf*, and *groEL*-*stamp*-*nadE*, as well as *rplO*-*secY*-*rpmJ* and *groEL* for ‘*Ca*. A. phytopathogenicus’. Amplicon pools underwent single-molecule real-time sequencing and amplicon-sequence-variant inference. Additionally, planthopper samples from sugar beet in Germany were analysed and compared to sugar beet data for ‘*Ca*. A. phytopathogenicus’. No genetic diversity of ‘*Ca*. A. phytopathogenicus’ was detected, whereas 16SrXII-A and -P showed variation below the subgroup level. 16SrXII-A exhibited higher diversity than 16SrXII-P. In Germany, 16SrXII-A formed a single cluster, while 16SrXII-P comprised two clusters based on 16S rRNA-ITS-23S rRNA. In neighbouring countries, only 16SrXII-A showed diversity, resolving up to four clusters by *groEL*-*stamp*-*nadE*. These results provide a basis for the identification of dominant strains supporting comparative variety evaluation for tolerance.

## 1. Introduction

In Germany, sugar beet (*Beta vulgaris* subsp. vulgaris) is cultivated on approximately 380,000 hectares [1]. From 2020 to 2025, production averaged about 28.5 million tonnes of beet, corresponding to roughly 4.2 million tonnes of sugar per harvest campaign. This places Germany among the leading producers in Europe, where approximately 15 million tonnes of sugar beet are produced [2]. Over the past two decades, the bacteriosis syndrome “basses richesses” (SBR) has become an increasing threat to sugar beet cultivation in Europe. The phloem-limited γ-proteobacterium ‘*Candidatus* Arsenophonus phytopathogenicus’ represents the causative agent of SBR. However, since the beginning of the SBR outbreaks in 1991 in eastern France (Burgundy), besides ‘*Ca*. A. phytopathogenicus’, the sporadic presence of the bacterial stolbur pathogen ‘*Candidatus* Phytoplasma solani’ subgroup 16SrXII-A of the class of Mollicutes was described in SBR-affected regions [3,4,5]. SBR-affected plants show yellowing and necrosis of older leaves, while younger leaves are lanceolate-shaped, and roots display deformations. Moreover, a brownish discolouration of their vascular system is observed, and the beets show a reduction of up to 5% in absolute sugar content and losses of up to 25% in overall biomass, making this disease economically significant. During the initial SBR outbreaks in France, severe yield losses were observed on approximately 1000 hectares of sugar beet production areas [6]. Its rapid spread potential was revealed early by an increase in SBR-affected area up to 1800 hectares in 2004 [7]. After the rapid spread of SBR reported in France, the first SBR outbreaks in Southern Germany near Heilbronn (Baden-Württemberg) were recognised in 2008 [8]. Later, in 2023, a ‘*Ca.* P. solani’-related stolbur pathogen assigned to a novel stolbur phytoplasma subgroup, designated 16SrXII-P, was identified in the Elbe river valley in eastern Germany (Saxony-Anhalt). There, the 16SrXII-P stolbur pathogen was associated with severe sugar beet losses and has been reported to occur in single and mixed infections with ‘*Ca*. A. phytopathogenicus’ [9].

Both ‘*Ca*. A. phytopathogenicus’ and the stolbur phytoplasmas of the subgroups 16SrXII-A and 16SrXII-P are transmitted by the cixiid vector *Pentastiridius leporinus* to sugar beet [3,4,9]. The vector was initially described as feeding on reed (*Phragmites australis*) in its natural habitat, before it was recognised as a pest of sugar beet [10]. Since *P*. *leporinus* was linked to the first SBR outbreak in the Heilbronn region in Germany [8], its population has increased enormously, accompanied by the rapid spread of the pathogens [11,12]. From 2023 to 2025, the estimated affected production reached 123,000 hectares, representing around one-third of the national sugar beet cultivation area [13]. In addition, *P*. *leporinus* has been observed in multiple crop systems in Germany since 2009, particularly within sugar beet–winter wheat (*Triticum aestivum*) rotations, as well as in various vegetable crops and maize (*Zea mays*) [8,11]. Recent studies and reports have further confirmed an expansion of its host range and the transmission of agents from both groups to economically important crops, including potato (*Solanum tuberosum*) [14,15,16], onion (*Allium cepa*) [17], and carrot (*Daucus carota* subsp. sativus) [18,19].

Hitherto, the bacterial pathogens have also been reported in sugar beet in other European countries apart from France and Germany. ‘*Ca*. A. phytopathogenicus’ has also been reported in sugar beet production areas in Switzerland, Austria, the Czech Republic, Slovakia, Hungary, Serbia, and Romania [20,21]. In Central Europe, ‘*Ca*. A. phytopathogenicus’ co-occurs mainly with ‘*Ca*. P. solani’ subgroup 16SrXII-A in sugar beet [21]. While in France and Germany, the subgroup 16SrXII-A is only sporadically present in sugar beet, in central Europe, this subgroup is associated as the main driver and causative agent for the rubbery taproot disease (RTD) that was recently described in Serbia [22]. RTD-affected plants show similar symptoms compared to SBR, which makes differentiation in the field quite challenging [22,23]. However, in contrast to France, Germany, and Switzerland, where *P*. *leporinus* is the primary vector associated with pathogen spread, multiple vector systems have been implicated in RTD epidemiology in sugar beet, including *Reptalus artemisiae* (formerly *Reptalus quinquecostatus*), *Reptalus panzeri*, and *Hyalesthes obsoletus* [24]. For Germany, the ‘*Ca*. P. solani’-related 16SrXII-P subgroup is considered as the key driver of the epidemic in sugar beet together with ‘*Ca*. A. phytopathogenicus’ [9,21,25], while in bordering countries 16SrXII-P is only present to a minor extent [21,26,27]. Moreover, actual findings from Austria showed that *R. artemisiae* represents the dominant RTD vector in this region and is also able to transmit all three pathogens to sugar beet [27]. This convergence of SBR- and RTD-associated pathogens and vector systems increases the complexity of effective disease management and highlights the need for robust molecular diagnostics and accurate differentiation frameworks.

**Figure 1:**
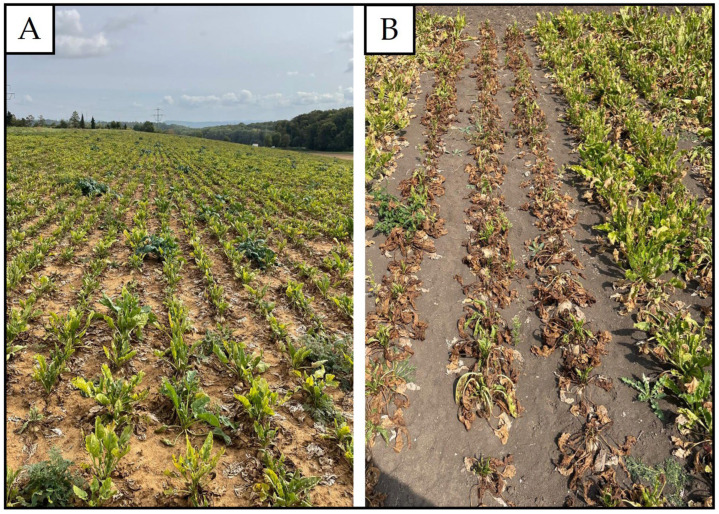
SBR-affected sugar beet plot (**A**) in Ditzingen (Baden-Württemberg, Germany). Severely affected sugar beet plants (**B**) from a field trial of different cultivars in Riedstadt-Leeheim (Rhineland-Palatinate, Germany).

Previous RTD and SBR studies have addressed pathogen occurrence and geographic spread, with strain-level diversity characterised largely for the 16SrXII-A subgroup in RTD-affected regions of central Europe [9,21,23,24,25]. In contrast, strain-level diversity remains unknown for ‘*Ca.* A. phytopathogenicus’ and the ‘*Ca*. P. solani’-related 16SrXII-P subgroup and is unresolved across German diseased areas. The limited resolution of strain-level diversity constrains epidemiological analyses of SBR-affected production areas (Figure 1A), including the identification of specific pathogen–vector relationships and regional transmission patterns as described for 16SrXII-A in the context of RTD in central Europe [24,28]. Furthermore, even the evaluation of different sugar beet cultivars within plot trials (Figure 1B) is already a focus in SBR research and the sugar industry [18]; strain-level information is crucial for effective breeding strategies and would improve the selection and development of tolerant cultivars.

Therefore, in this study, we employed long-read single-molecule real-time (SMRT) amplicon sequencing to investigate marker-based genetic diversity of ‘*Ca*. A. phytopathogenicus’ in sugar beet from Germany and selected neighbouring countries and in associated *P*. *leporinus* populations from German production regions, while phytoplasma diversity was assessed exclusively in sugar beet from the same regions, aiming to resolve strain-level diversity and regional distribution patterns of both pathogen groups.

## 2. Results

### 2.1. Assessment of ‘Ca. A. phytopathogenicus’ and Phytoplasmas in Sugar Beet

Across all analysed 2479 DNA-template plates obtained from sugar beet root tips of individual plants in the two-year survey in 2023 to 2024 (Tables S1 and S2), the applied assays returned a total of 1963 positive results, with 586 (29.85%) for ‘*Ca*. A. phytopathogenicus’, 621 (31.64%) for phytoplasmas, and 756 (38.51%) with both (Table 1).

Among the 1925 samples originating from Germany, a total of 1596 tested positive with the barcoded primers, of which 322 (20.18%) were positive for ‘*Ca*. A. phytopathogenicus’ only and 523 (37.77%) for phytoplasmas, whereas 751 accounted for both pathogens (38.96%). Positive samples were recovered from five investigated German regions, including Heilbronn-Franconia, Stuttgart, Saxony-Anhalt/Brandenburg, Rhineland-Palatinate, and Donau-Isar, while no positive samples were observed for North Rhine-Westphalia. In addition, of the 554 samples from neighbouring countries, 367 tested positive, with 264 (71.94%) for ‘*Ca*. A. phytopathogenicus’, and 98 (26.70%) were associated with phytoplasmas only, while in five samples (1.36%), both pathogens were detected simultaneously. Taken together, five of the seven bordering countries provided positive samples, represented by Switzerland, France, Austria, Poland, and Slovakia. Samples from the Netherlands and Belgium provided no positive results. Overall, of the analysed sugar beet samples, most samples showed mixed infections, followed by single phytoplasma infection and those infected by ‘*Ca*. A. phytopathogenicus’.

### 2.2. Assessment of ‘Ca. A. phytopathogenicus’ in P. leporinus

Analyses of the planthopper samples focused on ‘*Ca*. A. phytopathogenicus’—the causative agent of SBR, for which, in contrast to phytoplasmas, even basic population structure-like evidence for distinct subgroups remains unresolved in sugar beet systems, complicating the identification of transmission patterns and motivating targeted analyses to improve their resolution. Out of the 240 planthopper samples, 194 (80.83%) samples tested positive for ‘*Ca.* A. phytopathogenicus’ in the screening with both marker loci (Table 2). Planthoppers tested from 2023 provided the overall lowest number of positive results, with 70.00% positive. In contrast, the region Heilbronn-Franconia showed the highest contribution with 97.50%, followed by Stuttgart and Saxony-Anhalt (90.00%), while Donau-Isar showed the lowest number of positives with 80% positives. Out of the 240 samples, 232 (96.67%) tested positive using the *P*. *leporinus*-specific conventional PCR assay targeting the COI gene [29], whereas eight samples remained negative. These negative samples comprised five from the Heilbronn-Franconia region collected in 2023 and three from Rhineland-Palatinate collected in 2025.

### 2.3. SMRT-Sequencing Results

Sequencing of approximately 4.66 µg of equimolar pooled amplicon DNA yielded a total of 11,209,515 unfiltered reads (Table S3), representing 5932 positives (Table 3). Of these 3,450,874 reads (30.79%) were obtained from ‘*Ca*. A. phytopathogenicus’-targeted amplicons, comprising 1,728,876 reads for the *rplO*-*secY*-*rpmJ* target and 1,721,998 reads for *groEL*, with an average length of 1459 nt and 1559 nt, respectively. Amplicons generated using phytoplasma-targeted amplicons contributed 7,758,641 reads (69.21%), including 3,875,213 reads for the 16S rRNA-ITS-23S rRNA region, 1,522,102 reads for *tuf*, and 2,361,326 reads for the *groEL-stamp-nadE* region. All reads showed an average quality score of 39.7. Read data of the phytoplasma targets (16S rRNA-ITS-23S rRNA, *tuf*, and *groEL-stamp-nadE*) showed an average length of 1745 nt, 1046 nt, and 2865 nt, respectively.

### 2.4. Diversity Analysis

#### 2.4.1. Assignment of Amplicon Sequence Variants (ASVs)

Following quality and length filtering, a total of 5,268,798 reads remained in the data set (Table 3), of which 1,643,393 reads (31.20%) were associated with the ‘*Ca*. A. phytopathogenicus’ markers, with 877,107 reads for *rplO*-*secY*-*rpmJ* and 766,286 reads for *groEL*. ASV reconstruction of both markers produced consistently one ASV across sugar beet and *P*. *leporinus* (Tables S4 and S5). For phytoplasmas, a total of 3,625,405 (68.80%) reads remained in the data set, with 1,578,874 of 16S rRNA-ITS-23S rRNA representing 14 ASVs (Table S6), 972,009 for *tuf* building 18 ASVs (Table S7), and 1,074,522 reads as part of the *groEL-stamp-nadE* data forming 14 ASVs (Table S8).

Basic local alignment searching tool (BLAST) v2.16 analysis [30], for the further selection of target-specific ASVs of the ‘*Ca*. A. phytopathogenicus’ markers, confirmed target specificity and showed no differences to the draft genome sequence strains UHOH [31], Ap-CH and Ap-FR [32], as well as to the complete genome sequences of ‘*Ca*. A. phytopathogenicus’ strain PENLEP [33]. BLAST analysis of the predicted phytoplasmas ASVs of the 16S rRNA-ITS-23S rRNA ASVs enabled the differentiation of the two stolbur phytoplasma subgroups 16SrXII-A and -P, yielding seven ASVs assigned to 16SrXII-A and seven ASVs assigned to 16SrXII-P, and additionally identified one ASV belonging to the taxon ‘*Ca*. Phytoplasma asteris’ [34] subgroup 16SrI-A. Similarly, the *tuf* and *groEL-stamp-nadE* markers confirmed the presence of both stolbur subgroups, with *tuf* yielding three 16SrXII-A ASVs and 15 ASVs for 16SrXII-P, while *groEL-stamp-nadE* analysis produced five and nine ASVs for the same subgroups, respectively. Unlike the universal 16Sr rRNA-ITS-23S rRNA marker results, no ASVs of ‘*Ca*. P. asteris’ were detected with these markers, consistent with their group-specific primer design.

#### 2.4.2. Phylogenetic Clustering

**Figure 2:**
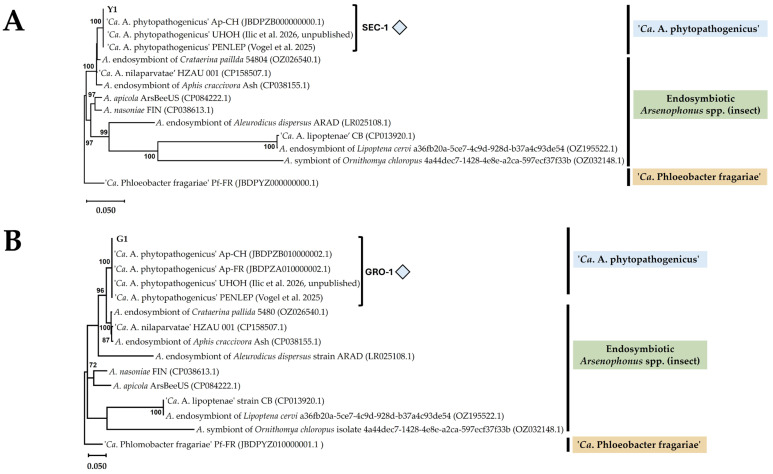
Maximum-likelihood trees of predicted ASVs of ‘*Ca*. A. phytopathogenicus’ (pale blue) markers *rplO*-*secY*-*rpmJ* (**A**) and partial *groEL* (**B**), with selected sequences from complete reference genomes of endosymbiotic *Arsenophonus* spp. (pale dark green) and ‘*Ca*. Phloeobacter fragariae’ (pale brown). Trees were reconstructed from multiple sequence alignments comprising 1468 and 1554 nucleotide positions, respectively, and inferred using the maximum-likelihood approach with 1000 bootstrap replicates. Bootstrap support values ≥ 70% are shown at the corresponding nodes. Branch lengths are drawn to scale and represent the number of substitutions per site. Accession numbers/references of reference strains are given in parentheses. ASV names are highlighted in bold; clusters from sugar beet and *P*. *leporinus* are indicated with a rhomb and summarised with a curly bracket.

Phylogenetic clustering of the single ASVs Y1 and G1 from sugar beet and *P*. *leporinus* of ‘*Ca*. A. phytopathogenicus’ with reference data formed each one cluster (SEC-1 and GRO-1) together with the reference draft genome strains UHOH, Ap-CH, Ap-FR and the complete genome strain PENLEP (Figure 2) reconstructed from *P*. *leporinus*. Further, the ‘*Ca*. A. phytopathogenicus’ cluster was separated from the obtained endosymbiotic *Arsenophonus* species and ‘*Candidatus* Phloeobacter fragariae’ strain Pf-FR [35,36].

**Figure 3:**
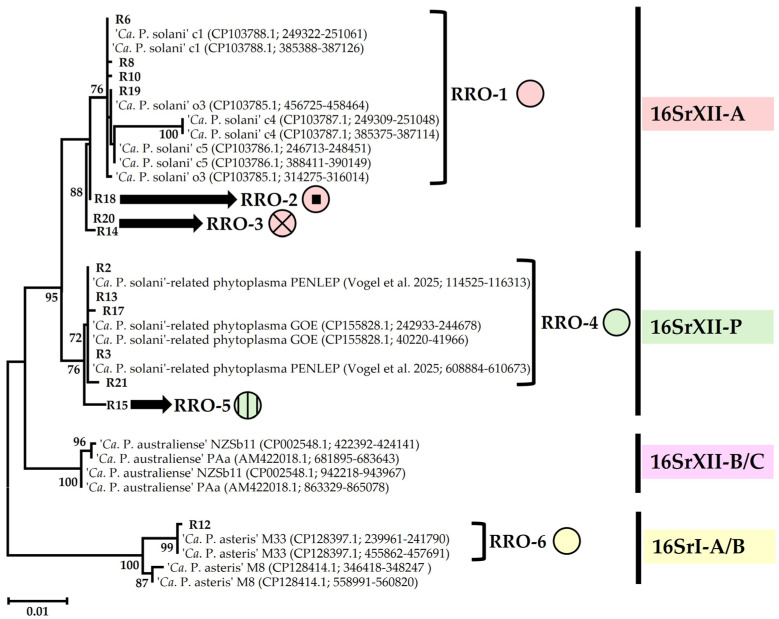
Maximum-likelihood tree of predicted ASVs (R2–21) and selected complete reference genomes of the stolbur group (16SrXII) and asteris group (16SrI) of ‘*Ca*. Phytoplasma’, based on the 16S rRNA-ITS-23S rRNA marker. For tree reconstruction, a multiple sequence alignment comprising 1797 nucleotide positions was utilised and inferred with 1000 bootstrap replicates. Bootstrap support values ≥ 70% are shown at the corresponding nodes. Branch lengths are drawn to scale and represent the number of substitutions per site. Accession numbers/references and the genomic positions of multiple copies in the reference strains are given in parentheses. ASV names are highlighted in bold, clusters from sugar beet are indicated by a circle and summarised either with a curly bracket or indicated by a black arrow. Colours indicate subgroup association with 16SrXII-A (pale red), 16SrXII-P (pale green), 16SrXII-B/C (pale purple), 16SrI-A/B (pale yellow). Different fill patterns of the circles indicate distinct sequence clusters within the subgroup.

Maximum-likelihood of the 14 ASVs with the phytoplasma reference genomes showed for the universal 16S rRNA-ITS-23S rRNA marker a separation of six ribosomal RNA operon (RRO) clusters (Figure 3). Three clusters were assigned to the 16SrXII-A subgroup of stolbur phytoplasmas, namely RRO-1, comprising four ASVs; RRO-2 (one ASV); and RRO-3 (two ASVs), whereas for 16SrXII-P, two clusters were separated and named RRO-4 (nine ASVs) and RRO-5 (one ASV). Initial phylogenetic clustering showed that the RRO-1 line included complete reference genomes of ‘*Ca*. P. solani’ strains c1, c4, c5, and o3 [37], whereas lines RRO-2 and RRO-3 were separated individually. Within the 16SrXII-P subgroup, RRO-4 represented the cluster associated with the complete genome sequence of the 16SrXII-P stolbur phytoplasma strain GOE [38] and the previously added 16SrXII-P strain PENLEP [33], while RRO-5 represented a different clade. Further, the single ‘*Ca*. P. solani’ cluster (RRO-6) was assigned to the 16SrI-A subgroup with the strain M33 [39].

**Figure 4:**
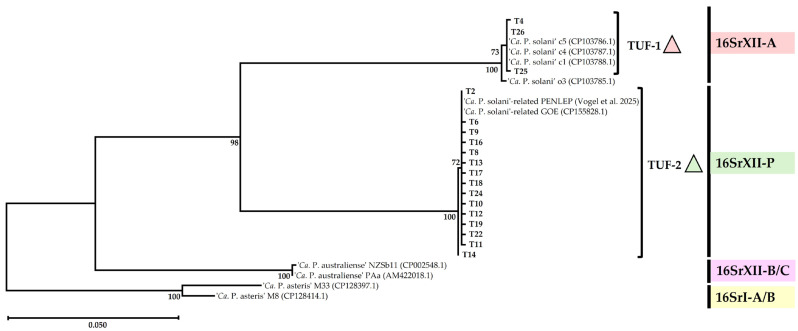
Maximum-likelihood trees of predicted ASVs (T2–T26) and selected complete reference genomes of the stolbur group (16SrXII) and asteris group (16SrI) of ‘*Ca*. Phytoplasma’, based on the partial gene *tuf*. Tree reconstruction was performed with a multiple sequence alignment comprising 1044 nucleotide positions and was inferred with 1000 bootstrap replicates. Bootstrap support values ≥ 70% are shown at the corresponding nodes. Branch lengths are drawn to scale and represent the number of substitutions per site. Accession numbers/references of reference strains are given in parentheses. ASV names are highlighted in bold, clusters from sugar beet are indicated by a triangle and summarised either with a curly bracket or indicated by a black arrow. Colours highlight subgroup association with 16SrXII-A (pale red), 16SrXII-P (pale green), 16SrXII-B/C (pale purple), 16SrI-A/B (pale yellow).

Analysis of the 18 *tuf* ASVs revealed a separation of two clusters (Figure 4), namely, TUF-1 (three ASVs) and TUF-2 (15 ASVs), representing the subgroups 16SrXII-A and -P, while o3 also indicated separation within the obtained cluster. A finer separation within the 16SrXII-P subgroup cluster TUF-2 was also observed for ASV T14. However, due to the very low level of sequence polymorphisms, the potential influence of amplification and sequencing errors, and the limited availability of biologically relevant reference data for different *tuf* lineages from sugar beet within 16SrXII-P, this divergence was considered insufficient to justify the definition of a new cluster. T14 was therefore retained within the TUF-2 cluster.

**Figure 5:**
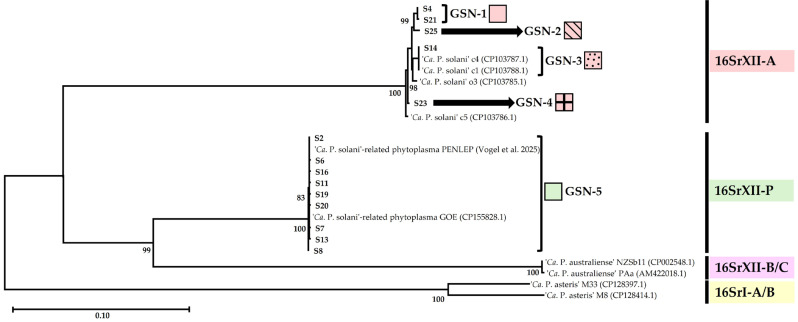
Maximum-likelihood trees of predicted ASVs (S2–S25) and selected complete reference genomes of the stolbur group (16SrXII) and asteris group (16SrI) of ‘*Ca*. Phytoplasma’, based on the marker region *groEL*-*stamp*-*nadE*. Tree construction comprised a multiple sequence alignment with a total of 3554 nucleotide positions and was inferred with 1000 bootstrap replicates. Bootstrap support values ≥ 70% are shown at the corresponding nodes. Branch lengths are drawn to scale and represent the number of substitutions per site. Accession numbers/references of the reference strains are given in parentheses. ASV names are highlighted in bold, clusters from sugar beet are indicated by a square and summarised either with a curly bracket or indicated by a black arrow. Colours indicate subgroup association with 16SrXII-A (pale red), 16SrXII-P (pale green), 16SrXII-B/C (pale purple), 16SrI-A/B (pale yellow). Different fill patterns of the squares indicate distinct sequence clusters within the subgroup.

Further, the evaluation of the 14 *groEL-stamp-nadE* ASVs led to the differentiation of four genetic clusters (Figure 5) of the 16SrXII-A subgroup, namely, GSN-1 (two ASVs), GSN-2 (one ASV), GSN-3 (one ASV), and GSN-4 (one ASV), and a separation of the strains c5 and o3. Within the 16SrXII-P subgroup, most sequences grouped into a dominant cluster referred to as GSN-5 (nine ASVs). Although one ASV (S8) showed a separation from this cluster, the divergence was restricted to a maximum of two nucleotide polymorphisms and considered insufficient to justify the definition of a new cluster, as described for *tuf*.

#### 2.4.3. Abundances of ASV Clusters

Of all analysed markers, ASV clustering revealed a strong dominance of single clusters for each target organism (Table 4). For ‘*Ca*. A. phytopathogenicus’, all passed reads obtained for the *rplO*-*secY*-*rpmJ* and *groEL* markers clustered exclusively into SEC1 and GRO1, respectively, each accounting for 100% of reads in both sugar beet and *P*. *leporinus*.

For phytoplasmas, the 16S rRNA-ITS-23S rRNA marker passed read data was dominated by clusters assigned to the 16SrXII-P subgroup, with RRO-4 representing the major cluster (>90% of reads), while RRO-5 was only present to a minor extent (<0.1%). In contrast, the 16SrXII-A subgroup clusters (RRO-1 to RRO-3) showed low abundances with less than ten percent. Further, the single 16SrI-A cluster (RRO-6) also represented a low-abundance cluster. Similarly, *tuf* and *groEL-stamp-nadE* markers showed a pronounced dominance of 16SrXII-P-associated clusters, with TUF-2 and GSN-5 accounting for more than 99% of reads, respectively. Minor clusters affiliated with the 16SrXII-A subgroup were present at low relative abundances across both markers.

#### 2.4.4. Sequence Variation Analysis on Nucleotide Level

Sequence difference analysis of the ‘*Ca*. A. phytopathogenicus’ markers *rplO*-*secY*-*rpmJ* (Table S9) and *groEL* (Table S10) was based on multiple sequence alignments comprising 1468 and 1554 positions, respectively, and confirmed clustering with 100% sequence identity. As no subgroups could be separated and only a single ASV cluster was detected per marker (SEC-1 and GRO-1), inter-subgroup and inter-cluster identities were not applicable (Table 5). Further, both clusters were separated from the nine reference genomes of the endosymbiotic *Arsenophonus* spp., with identities ranging from 80.3 to 90% for *rplO*-*secY*-*rpmJ* and 83.5 to 97.1% for *groEL*. ‘*Ca*. Phloeobacter fragariae’ Pf-FR [32] showed 95.2% identity to ‘*Ca*. A. phytopathogenicus’ for the cluster SEC-1, and 90.5% for GRO-1.

Sequence variation analysis of the phytoplasma marker 16S rRNA-ITS-23S rRNA was based on an alignment of 1797 nucleotide positions (Table S11). Sequence identity between the stolbur subgroups 16SrXII-A and 16SrXII-P ranged from 97 to 99% (Table 5). The 16SrXII-A subgroup clusters (RRO-1-RRO-3) showed the most difference within the 16SrRNA, while the 16SrXII-P clusters RRO-4-RRO-5 differed mainly in the 23S rRNA. Inter-cluster identity ranges within the subgroups were slightly higher, with approximately 98 to 99% for 16SrXII-A and ≥99% for 16SrXII-P. Intra-cluster sequence identities were also higher for 16SrXII-A, with approximately 98 to 100% identities, whereas 16SrXII-P showed a 99.7–100% range in lower variation within a cluster. The ‘*Ca*. P. asteris’ [34] subgroup 16SrI-A cluster (RRO-6) was clearly separated from the stolbur subgroups to 16SrXII-A and -P (92.6–93.9%), which was also the case for 16SrXII-B/C subgroups of the ‘*Ca*. P. australiense’ [40] with 96 to 97.8%.

For the *tuf* marker, sequence variation was assessed based on an alignment of 1044 nucleotide positions (Table S12). Inter-subgroup sequence identity between the 16SrXII-A and 16SrXII-P subgroups, represented by the ASV clusters TUF-1 and TUF-2, respectively, was approximately 89% (Table 5). Within each subgroup, intra-cluster identities were, in contrast, very high, with approximately 100% for both subgroups. Separation from other phytoplasma taxa was also underlined, with (86.1–87.6%) for ‘*Ca*. P. asteris’ and (87.3–89.1%) for ‘*Ca*. P. australiense’.

For the *groEL-stamp-nadE* locus, sequence variation was analysed based on an alignment of 3554 positions (Table S13). Identities between the subgroups 16SrXII-A and -P ranged from 73.1 to 73.9% (Table 5). While in 16SrXII-A subgroup clusters (GSN1-4), sequence variation was predominantly associated with the *stamp* gene; in 16SrXII-P, *groEL* showed higher, but still low sequence variation. Further, higher sequence variation in the 16SrXII-A subgroup was confirmed by inter-cluster identities (98.8–99.4%) that were not applicable for the single 16SrXII-P cluster (GSN-5), while intra-cluster sequence identities approached 100% for both subgroups. Separation of the taxa ‘*Ca*. P. australiense’ (61.8 to 69.9%) and ‘*Ca*. P. asteris’ (62.9–70.5%) were also highlighted.

#### 2.4.5. Impact of Sequence Variation on Amino Acid Level

After the investigation of sequence variation at the nucleotide level, coding sequences of the obtained markers that showed diversity were analysed to estimate the impact of non-synonymous changes on the amino acid level. For the marker region *rplO*-*secY*-*rpmJ of* ‘*Ca*. A. phytopathogenicus’, analysis was restricted to the complete coding sequence of *secY* (Table S14), since *rplO* and *rpmJ* code only for seven of 17 amino acids, which was considered too short for interpretation. In total, 441 amino acid positions were compared, whereas the *groEL* marker contributed 517 positions that were compared (Table S15). Both clusters from sugar beet and *P*. *leporinus* (SEC-1 and GRO-1) were clearly separated from the reference *Arsenophonus* spp., showing 4–70 variable nucleotide positions for *secY* and 11–55 for *groEL*. A similar level of divergence was observed for the phytopathogen ‘*Ca*. Phloeobacter fragariae’ strain Pf-FR (10 and 35 difference, respectively).

Comparison of the coding sequence of *tuf* ASVs involved 346 amino acid positions. Comparison of 16SrXII-A and -P subgroups showed a maximum of 22 variable amino acid positions (Table S16). Within the 16SrXII-A subgroup cluster (TUF-1), sugar beet ASVs showed no difference from each other and were identical to the reference strains c1, c4, and c5 from bindweed. In contrast, the stinging nettle strain o3 showed two non-synonymous exchanges, supporting its separation within the cluster. Within the 16SrXII-P cluster (TUF-2), only two of the 15 ASVs from sugar beet showed identical amino acid sequences in accordance with the reference strains GOE and PENLEP. A total of 13 ASVs all showed a single variation at different positions. The ‘*Ca*. P. australiense’ and ‘*Ca*. P. asteris’ strains were separated as indicated on a nucleotide basis with 17–24 positions and 14–29, respectively.

Amino acid alignments of the coding regions for the marker *groEL*-*stamp*-*nadE* were based on 438 positions for partial *groEL*, 249 for the complete *stamp* sequences, and 209 for the partial *nadE* sequence. Sequence differences between 16SrXII-A and 16SrXII-P subgroup variable positions for *groEL*, ranging from 50 to 52 (Table S17A), 98 to 105 for *stamp* (Table S17B)*,* and 37 to 39 for *nadE* (Table S17C), also confirmed subgroup differentiation at the amino acid level. Higher variation in the 16SrXII-A subgroup was also confirmed with an inter-cluster variation in two for *groEL*, 4–20 for *stamp*, and only one variable position for the amino acid sequence of *nadE*. Strain o3 was also separated by showing the highest variation, followed by c5. In contrast, within the sugar beet clusters, no sequence variation was observed for the 16SrXII-A subgroup. Within the 16SrXII-P subgroup, *groEL* showed the maximum of two variable positions within the GSN-5 cluster, while only one amino acid substitution for *stamp* and *nadE* was identified. Separation of the ‘*Ca*. P. australiense’ and ‘*Ca*. P. asteris’ strains was also confirmed across all three coding sequence targets. Therefore, investigations at the amino acid level supported those observed from clustering with nucleotide sequences.

#### 2.4.6. Comparison with Reference Data from Sugar Beet

For the *rplO-secY-rpmJ* and *groEL* markers of ‘*Ca*. A. phytopathogenicus’, a direct comparison with sugar beet reference material was not feasible, as no comparable reference sequences from sugar beet are currently available. While no *groEL* sequences of this pathogen are deposited in the GenBank database of the National Center for Biotechnology Information (NCBI), a single *secY* sequence from onion (*Allium cepa*) (PP950433.1) is available, which showed 100% pairwise identity to the obtained *secY* data from sugar beet and planthopper.

**Figure 6:**
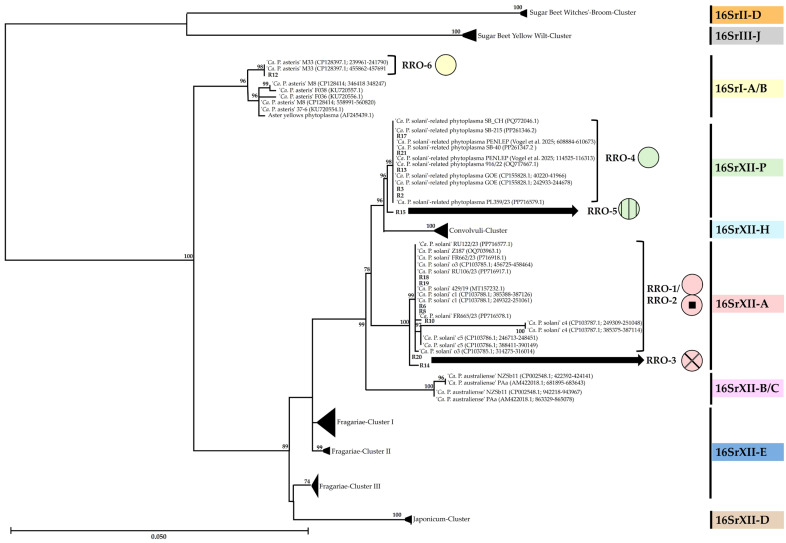
Maximum-likelihood trees of predicted ASVs (R2–R21) and sequences from sugar beet references and selected related stolbur/phytoplasma subgroups based on partial 16S rRNA gene sequence. The tree was reconstructed from a multiple sequence alignment comprising 1105 nucleotide positions and inferred with 1000 bootstrap replicates. Bootstrap support values ≥ 70% are shown at the corresponding nodes. Branch lengths are drawn to scale and represent the number of substitutions per site. Accession numbers/references and the genomic positions of multiple copies in the reference strains are given in parentheses. Associated genotypes from sugar beet are grouped and indicated with the predicted clusters (circles) from full-length marker analyses with curly brackets and arrows. Colours indicate subgroup association with 16SrIII-D (pale orange), 16SrIII-J (grey) as outgroup, 16SrI-A/B (pale yellow), 16SrXII-P (pale green), 16SrXII-H (pale light blue), 16SrXII-A (pale red), 16SrXII-B/C (pale purple), 16SrXII-E (pale dark blue), 16SrXII-D (pale brown). Sequences included in the given clusters (triangles) are given in the Supplementary Materials (Table S18). Different fill patterns of the circles indicate distinct sequence clusters within the subgroup.

For the phytoplasmas, comparison of the 16S rRNA-ITS-23S rRNA sequences with publicly available sugar beet reference data confirmed the assignment of clusters RRO-1 to RRO-3 to the 16SrXII-A subgroup, as well as that of RRO-4 and RRO-5 to the 16SrXII-P subgroup of the stolbur group (16SrXII), while RRO-6 grouped with the 16SrI-A subgroup of ‘*Ca*. P. asteris’ (Figure 6).

**Figure 7:**
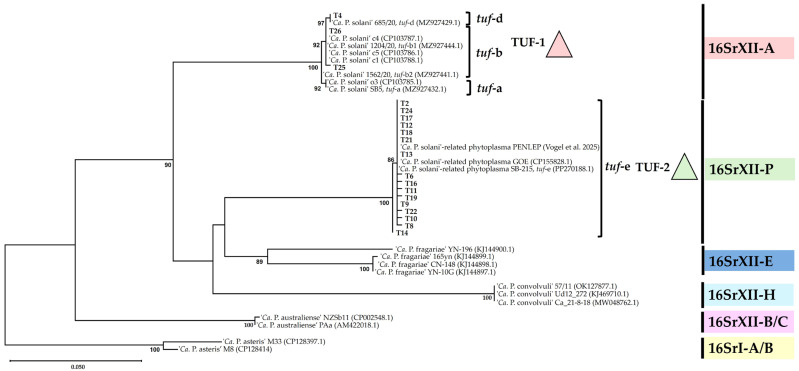
Maximum-likelihood trees of predicted ASVs (T2–T26) and sequences from sugar beet references and selected related stolbur/phytoplasma subgroups based on partial *tuf*. Trees were reconstructed from a multiple sequence alignment with 598 nucleotide positions and inferred with 1000 bootstrap replicates. Bootstrap support values ≥ 70% are shown at the corresponding nodes. Branch lengths are drawn to scale and represent the number of substitutions per site. Accession numbers/references of the reference strains are given in parentheses. Associated genotypes from sugar beet are grouped and indicated with the predicted clusters (triangles) from full-length marker analyses with brackets. Colours indicate subgroup association with 16SrXII-A (pale red), 16SrXII-P (pale purple), 16SrXII-H (pale light blue), 16SrXII-E (pale dark blue), 16SrXII-B/C (pale green), and 16SrI-A/B (pale yellow) as outgroup.

Analysis of the marker *tuf* with selected genotypes from sugar beet provided additional information since the separation of two *tuf*-clusters, *tuf*-b and *tuf*-d, within the TUF-1 cluster associated with the 16SrXII-A subgroup was observed (Figure 7). For 16SrXII-P, the ASVs of the cluster TUF-2 showed clustering with the previously proposed line *tuf*-e [25].

**Figure 8:**
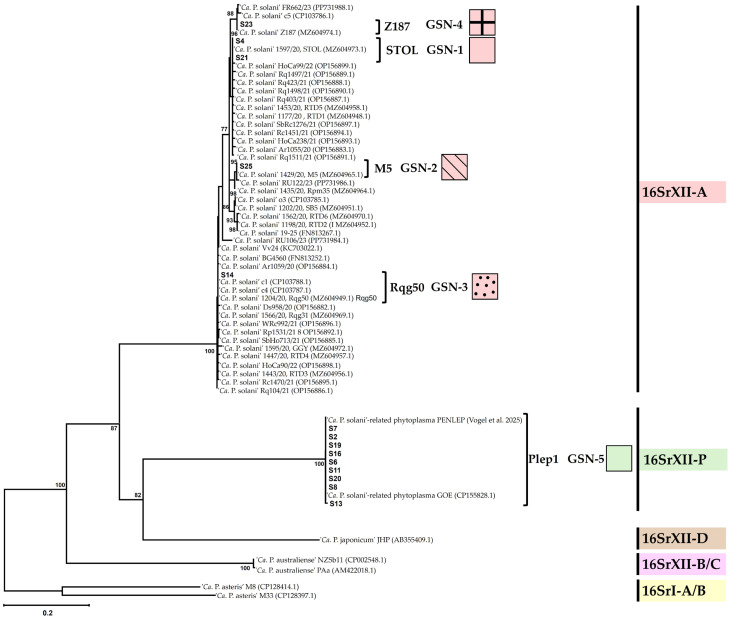
Maximum-likelihood trees of predicted ASVs (S2–S25) and sequences from sugar beet references and selected related stolbur subgroups based on the complete gene *stamp*. Trees were reconstructed from multiple sequence alignment comprising 773 nucleotide positions and inferred with 1000 bootstrap replicates. Bootstrap support values ≥ 70% are shown at the corresponding nodes. Branch lengths are drawn to scale and represent the number of substitutions per site. Accession numbers/references of the reference strains are given in parentheses. Associated genotypes from sugar beet are grouped and indicated with the predicted clusters (squares) from full-length marker analyses with curly brackets. Colours indicate subgroup association with 16SrXII-A (pale red), 16SrXII-P (pale green), 16SrXII-D (pale brown), 16SrXII-B/C (pale purple), 16SrI-A/B (pale yellow) as outgroup. Different fill patterns of the squares indicate distinct sequence clusters within the subgroup.

Comparison with previously described full-length *stamp* genotypes from sugar beet (Figure 8) confirmed the presence of five distinct clusters within the obtained sequence data from sugar beet. The 16SrXII-A group cluster GSN-1 showed the closest relation to genotype STOL (MZ604973.1), as well as GSN-2 to M5 (MZ604960.1), whereas GSN-3 grouped with Rqg50 (KC703019.1), and GSN-4 with Z187 (MZ604974.1). BLAST analysis confirmed these assignments, showing 100% identity to the respective reference sequences. For 16SrXII-P, complete *stamp* genes from GSN-5 represented the same genotype found in sugar beet 16SrXII-P strains GOE and PENLEP. This corroborates previous findings analysing 16SrXII-P *stamp* genotypes occurring in German sugar beet fields, which were suggested to be associated with *P*. *leporinus* [25]. The 16SrXII-P *stamp* genotype is therefore hereafter referred to as Plep1.

Overall, these results reveal that the markers *rplO*-*secY*-*rpmJ* and *groEL* of ‘*Ca*. A. phytopathogenicus’ could not differentiate clusters below the species level in sugar beet and *P*. *leporinus* samples but clearly separated them from other known endosymbiotic *Arsenophonus* spp. from insects and the related phloem parasite, ‘*Ca*. Phloeobacter fragariae’. Analysis of the phytoplasma markers 16S rRNA-ITS-23S rRNA, *tuf*, and *groEL-stamp-nadE* showed the presence of three phytoplasma subgroups in the analysed sugar beet samples, with the two stolbur subgroups 16SrXII-A and -P, as well as the subgroup 16SrI-A of the taxon ‘*Ca*. P. asteris’, which is in accordance with previous findings [21]. Further, the minor present 16SrXII-A subgroup showed higher genetic diversity compared to the dominating 16SrXII-P subgroup. For 16SrXII-A, the highest genetic resolution was achieved using *groEL-stamp-nadE*, while for 16SrXII-P finest separation was achieved by the ribosomal operon. Therefore, our marker analysis confirms the presence of previously described phytoplasmas in sugar beet and provides additional information on 16SrXII-P diversity based on ribosomal operon variation.

### 2.5. Regional Occurrence of Predicted ASV Clusters

**Figure 9:**
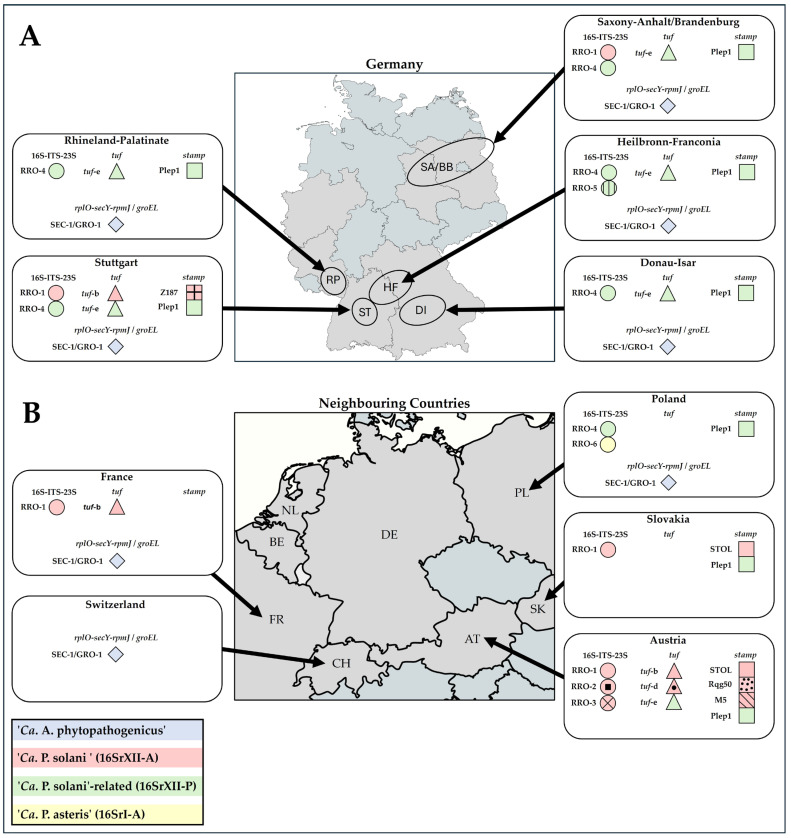
Schematic overview of the regional distribution of the predicted ASV clusters/genotypes from ‘*Ca*. A. phytopathogenicus’ with *rplO*-*secY*-*rpmJ* and *groEL* (rhombs*)* and the phytoplasma markers 16S rRNA-ITS-23S rRNA (circles), *tuf* (triangles*)*, and *stamp* (squares) within Germany (**A**), and neighbouring countries (**B**). Schematic maps were created and modified from MapChart (https://mapchart.net). Colours indicate association with ‘*Ca*. A. phytopathogenicus’ (pale blue) and phytoplasmas of the subgroup 16SrXII-A (pale red), 16SrXII-P (pale green), 16SrXII-B/C (pale purple), and 16SrI-A/B (pale yellow). Federal states of Germany and neighbouring countries, from which the sampling regions were derived, are highlighted in pale grey.

The ASV clusters for ‘*Ca*. A. phytopathogenicus’ (GRO-1, SEC-1) were both found in all five German regions analysed (Figure 9). Moreover, the neighbouring countries, France, Switzerland and Poland, also showed the presence of the clusters.

Regional distribution of ASV clusters based on the phytoplasma marker 16S rRNA-ITS-23S rRNA marker showed that RRO-1 was the only 16SrXII-A cluster detected in Germany, occurring in Saxony-Anhalt and the Stuttgart region. RRO-1 was also detected in the neighbouring countries, France, Austria and Slovakia, whereas the clusters RRO-2 and RRO-3 were exclusively identified in Austria. 16SrXII-P cluster RRO-4 was found in all German regions, while cluster RRO-5 was only identified in Heilbronn-Franconia. Further, Poland was the sole region where RRO-4 was identified and additionally the only neighbouring country where the cluster RRO-6 of ‘*Ca*. P. asteris’ subgroup 16SrI-A was identified.

Regional analysis of the *tuf* data (Figure 9) from Germany confirmed 16SrXII-A presence only for Stuttgart with the *tuf*-b line (TUF-1 cluster). In the analysed neighbouring countries, *tuf*-b was confirmed for France and Austria, while within the Austrian samples, *tuf*-d was also detected. Moreover, *tuf* also supported omnipresence of the 16SrXII-P subgroup with *tuf*-e (TUF-2) being found in all German regions. Additionally, *tuf*-e was also identified in Austria.

For the *groEL-stamp-nadE* analysis of the German regions, 16SrXII-A occurrence was corroborated for Stuttgart only with the *stamp* genotype Z187 (GSN-4), whereas STOL (GSN-1) was identified in Austria and Slovakia (Figure 9). The 16SrXII-A *stamp* genotypes M5 (GSN-2) and Rqg50 (GSN-3) were assigned exclusively to Austria, while GSN-4 was present in Austria as well as in Slovakia. The *stamp* genotype Plep1 (GSN-5) of the 16SrXII- P subgroup was also found in all German regions and affirmed the observed omnipresence of the 16SrXII-P subgroup as demonstrated for the other markers. Further, Plep1 was also found in Poland, Austria and Slovakia.

Taken together, our analyses demonstrated the presence of ‘*Ca.* A. phytopathogenicus’ in sugar beet and its vector, *P. leporinus*, across all analysed German regions. In addition, its occurrence in sugar beet from neighbouring countries, including France and Switzerland, confirmed previous reports [4,20] and further extended the known geographic distribution of ‘*Ca.* A. phytopathogenicus’ to Poland. Phytoplasmas of the 16SrXII-A subgroup occurred only sporadically in German sugar beet but showed consistency of one genetic cluster across all markers with RRO-1, *tuf*-b and *stamp* genotype Z187, which confirms previous observations in German sugar beet plots. Furthermore, our data also supported 16SrXII-A dominance in central European countries, such as Austria and Slovakia [21,23]. Our data further support earlier suggestions that the 16SrXII-P subgroup represents the main driver of the SBR epidemic in German sugar beet cultivation areas [9,25], whereas 16SrXII-P is only present to a minor extent in the analysed bordering countries [21,26,27]. Furthermore, 16SrI-A phytoplasmas assigned to ‘*Ca.* P. asteris’ were confirmed in sugar beet from Poland, consistent with earlier findings [21,41].

## 3. Discussion

For the ‘*Ca*. A. phytopathogenicus’ markers *rplO*-*secY*-*rpmJ* and *groEL*, diversity analyses revealed a highly uniform pattern across all investigated samples and regions in both sugar beet and *P*. *leporinus* collected from sugar beet. However, both markers were well-suited for reliable detection and species-level differentiation but are not expected to resolve fine-scale population structure in such a genetically homogeneous taxon. This is in accordance with earlier work that tried to characterise ‘*Ca*. A. phytopathogenicus’. Available molecular markers for characterisation are scarce, and previous studies have mainly focused on ribosomal RNA operons [35,42], while non-ribosomal targets have been analysed using the *spoT*-*spoU*-*recG* region, encoding a (p)ppGpp hydrolase/synthetase, an RNA methyltransferase, and a DNA helicase [43]. However, both targets offer limited discriminatory power, and the multicopy nature of the rRNA operons can further confound diversity analyses due to their described interoperon heterogeneity [42]. Moreover, additional markers such as *manA* coding for the mannose-6-phosphate-isomerase and *tuf* (elongation factor Tu) have been described but remain insufficiently evaluated for strain-level differentiation [16,17].

Such limited sequence variation may be explained by a genetic bottleneck of the pathogen population, as a product of strong host associations with *P. leporinus* as the dominant vector of the pathogen in sugar beet, which completes its entire life cycle within sugar beet [44]. However, *P*. *leporinus* has expanded its host range to other economically important crops, such as potato [14,15,16] and carrot [18,19], as well as other vectors, like the RTD vector *R*. *artemesiae*, that are reported to carry and transmit the pathogen [27]. Such differing host environments and the involvement of additional vectors may contribute to altered selection pressures and potentially to further genetic diversification of the pathogen, as is also described for stolbur phytoplasmas, where epidemiological patterns are correlated with different plant hosts associated with the vector lifecycle [24,28,45,46]. Furthermore, since the beginning of this study, available sequence resources for ‘*Ca*. A. phytopathogenicus’ have increased but remain limited to one complete and two draft genomes reconstructed from *P*. *leporinus* [32,33], as well as one draft genome obtained from *R*. *artemesiae* [47]. Genomes or validated markers for strain-level characterisation from sugar beet or other associated host plants and insects are still lacking, which underlines the need for such data as a basis for the evaluation of fitting loci for characterisation to improve the understanding of the pathogen evolution of ‘*Ca*. A. phytopathogenicus’ in relevant cropping systems.

Stolbur phytoplasma analyses revealed genetic diversity within both subgroups, 16SrXII-A and 16SrXII-P. Analyses based on the 16S rRNA-ITS-23S rRNA marker resolved three sequence variant clusters within the 16SrXII-A subgroup (RRO-1, RRO-2, and RRO-3) and two for the 16SrXII-P subgroup (RRO-4 and RRO-5). While for the 16SrXII-A subgroup, differences were mainly located in the 16S rRNA region, and 16SrXII-P clusters differed mainly in the 23S rRNA region. Previous studies on phytoplasma classification based on ribosomal operon sequences already highlighted the phylogenetic potential of the 23S rRNA gene for phytoplasma group differentiation, but also pointed to the limited resolution at the subgroup level of this marker region [48,49]. Interestingly, our findings indicated that the 23S rRNA region may provide additional resolution for intra-subgroup differentiation within the 16SrXII-P subgroup, which should be considered for further studies for the characterisation of this subgroup. However, despite the fact that intra-operon heterogeneity within single strains has been described for stolbur phytoplasmas [50,51], it remains unclear whether the detected sequence variants represent epidemiologically relevant new lineages or heterogeneity that is not resolved by the currently available complete genome data for the 16SrXII-A [37,47,52] and 16SrXII-P subgroups [33,38], and therefore needs further confirmation at the single-sample level.

Analysis of the non-ribosomal markers *tuf* and *groEL-stamp-nadE* confirmed higher diversity for the 16SrXII-A subgroup in sugar beet with two *tuf* lineages (*tuf*-b and *tuf*-d) and four stamp genotypes (STOL, M5, Rqg51, Z187). While for 16SrXII-P, only a single cluster for each marker (*tuf*-e, Plep1) was identified. Therefore, our analysis corroborates previously described stolbur lines occurring in European sugar beet plots [21,22,23,25] and associated insect vectors [24,28]. Interestingly, in contrast to the 16SrXII-A subgroup, 16SrXII-P showed lower genetic diversity in the non-ribosomal targets compared to the ribosomal RNA operon. This may support the theory of a genetic bottleneck as a product of a highly specialised population, as speculated for ‘*Ca*. A. phytopathogenicus’ above. Further, this would be plausible since both pathogens are described to occur often in mixed infections [9], which is also supported by our data. In contrast to ‘*Ca*. A. phytopathogenicus’, such a vector-driven specialisation is known for stolbur phytoplasmas associated with the Bois noir-affected vineyards in Tuscany (Italy), where a single *stamp* genotype (St10) dominated the ‘*Ca*. P. solani’ population and was associated with a transmission cycle involving *R*. *artemesiae* rather than the commonly reported vector *H*. *obsoletus* [53].

Consistent with the observed patterns in sugar beet described, our data revealed pronounced regional differences in pathogen occurrence. In Germany, our data showed the presence of ‘*Ca*. A. phytopathogenicus’ and 16SrXII-P phytoplasmas in all five SBR regions analysed, whereas for phytoplasmas we found phytoplasmas of the 16SrXII-A subgroup only in the regions of Stuttgart and Saxony-Anhalt/Brandenburg. In total, one 16SrXII-A cluster with RRO-1, *tuf*-b and the *stamp* genotype Z187 was found, whereas for 16SrXII-P, two patterns were identified with RRO-4, *tuf*-e, and Plep1 and were found in all regions; as a second pattern, RRO-5 was only present in Heilbronn-Franconia. Therefore, the regional distribution of our data provides further confirmation that ‘*Ca*. A. phytopathogenicus’ and the ‘*Ca*. P. solani’-related stolbur phytoplasmas of the 16SrXII-P subgroup drive the SBR epidemic in Germany, while the subgroup 16SrXII-A plays a minor role in sugar beet, as indicated by other regional studies from eastern, western and southern Germany [9,23,25].

Investigation of the data obtained from neighbouring countries confirmed the presence of ‘*Ca*. A. phytopathogenicus’ in France and Switzerland [4,20] and added Poland as an additional region. Furthermore, our analysis corroborated previous reports from France, Austria, Slovakia, and Poland, identifying the 16SrXII-A subgroup of ‘*Ca.* P. solani’ as the predominant stolbur phytoplasma in these countries, while our data supports the latest reports of 16SrXII-P from Austria and Poland [21,27] and indicates the presence in Slovakia. This fits the observed situation in the Austrian RTD context of sugar beet, where *P*. *leporinus*, carrying both the SBR pathogens, ‘*Ca*. A. phytopathogenicus’ and the ‘*Ca*. P. solani’-related 16SrXII-P phytoplasmas, was reported, and their integration via *P*. *leporinus* was suggested along the sugar beet cultivation areas of the Danube basin [27]. From a geographical point of view, our data support this possibility, given the fact that results from Germany showed the presence of the pathogens in the region Donau-Isar, as well as shown by the first study that reported 16SrXII-P in Bavaria earlier [9]. Another integration from Switzerland would also be reasonable, since the pathogens and the vector *P*. *leporinus* were reported in sugar beet and potato previously [20,26,32,54]. Notably, in Austria, it was shown that the dominant RTD vector *R*. *artemesiae* is also able to carry and transmit both ‘*Ca*. A. phytopathogenicus’ and 16SrXII-P phytoplasmas to sugar beet besides 16SrXII-A phytoplasma [27]. Therefore, the detection of 16SrXII-P phytoplasmas in Slovakian sugar beet may be linked to the transmission of the pathogens to the main RTD vector, *R. artemisias*, which could facilitate the spread of this novel stolbur lineage from Austria to other regions of the Pannonian Plain. However, it still remains elusive whether the SBR vector *P*. *leporinus* has already arrived in the other countries facing RTD epidemics. Beyond this, the impact of other co-occurring cixiid vectors, in the context of 16SrXII-P spread, is still unknown, as well as the lack of information on natural reservoir plants for the 16SrXII-P subgroup, which are both crucial to understanding the spread of this novel stolbur pathogen and its vector epidemiology. Moreover, the confirmed overlap of SBR and RTD in neighbouring countries underlines the complexity of disease management, as highlighted by previous studies [21,27] and emphasises the need for high-throughput approaches capable of reliably differentiating the pathogens in sugar beet and their insect vectors.

## 4. Materials and Methods

### 4.1. Sugar Beet Samples

For diversity assessment of the pathogens in 2023, sugar beet root tips from individual plants were collected during the harvesting period from September to December 2023, randomly from sugar beet clamps (Table 6). Samples utilised tested positive either for ‘*Ca*. A. phytopathogenicus’ or phytoplasmas by the application of real-time PCR using a TaqMan assay for universal phytoplasma detection targeting the 16S rRNA gene [55] and ‘*Ca*. A. phytopathogenicus’-targeting gene *manA* [56]. This set of pre-selected positive samples (Table S1) encompassed 1085 samples identified from a total of approximately 1400 samples taken from five German regions, namely, Stuttgart, Heilbronn-Franconia, Donau-Isar, Saxony-Anhalt and Rhineland-Palatinate, as well as a subset of 347 positive samples identified from about 1200 samples from the six neighbouring countries: France, Switzerland, Austria, Poland, the Netherlands, and Belgium.

In 2024, the sampling strategy shifted towards exclusively collecting root tips from individual sugar beets from symptomatic sugar beet plants showing SBR-/RTD-like symptoms in the field, which were included in the analysis without prior real-time PCR screening (Table S2). Sampling covered a total of 1020 samples, of which 840 were from the same German regions as in 2023, with the Saxony-Anhalt region expanded to Brandenburg and with the additional region of North Rhine-Westphalia to account for the spread of pathogens. Further, a total of 180 samples were also added for the same neighbouring countries, with Slovakia included as an additional location.

### 4.2. Planthopper Samples

For the assessment of the diversity of ‘*Ca*. A. phytopathogenicus’ in 2023, individual samples of 40 planthoppers from sugar beet from the region of Heilbronn-Franconia were collected using sweeping nets from July to August. Additionally, 200 planthopper catches were added in 2025 from the five German sampling regions of Stuttgart, Heilbronn-Franconia, Donau-Isar, Saxony-Anhalt, and Rhineland-Palatinate during the growing season from May to September (Table 7).

### 4.3. Nucleic Acid Extraction from Sugar Beet and Planthopper Samples

For the extraction of nucleic acids from sugar beet, one gram of taproot tissue was homogenised in plastic extraction bags using a HOMEX 7 homogeniser (Bioreba AG, Grenzach, Switzerland) together with 4 mL lysis buffer of the High Pure PCR Template Preparation Kit (Roche Diagnostics GmbH, Mannheim, Germany). Extraction of the planthopper samples was conducted with whole individuals and 800 µL of the lysis buffer and was homogenised with five silica beads at 8 m/s for 90 s in a FastPrep-24 Instrument (MP Biomedicals, Eschwege, Germany). Subsequent extraction steps for both sugar beet and planthoppers were conducted according to the manufacturer’s instructions. Remaining macerates and purified nucleic acid extracts were stored at −20 °C for further analysis.

### 4.4. Marker Selection and Primer Design

For the development of endpoint PCR assays for regional diversity assessment, marker sequences were selected based on genome data of ‘*Ca*. A. phytopathogenicus’ and phytoplasmas occurring in the SBR-affected regions. For ‘*Ca*. A. phytopathogenicus’, a high-accuracy draft genome sequence of the strain UHOH [31] and the complete genome sequence of strain PENLEP [33] (https://doi.org/10.17617/3.4JXKRW) were used as marker databases, while the complete genome sequences of the ‘*Ca*. P. solani-related’ 16SrXII-P subgroup strains GOE (CP155828.1) [38] and PENLEP [33] (https://doi.org/10.17617/3.4JXKRW) served the same purpose for phytoplasmas. As a key selection criterion for new marker regions, only single-copy genomic regions capable of generating long, informative amplicons were considered. Candidate regions were compared to reference sequences of the NCBI database using the basic local alignment searching tool (BLAST) [30] against the nucleotide collection (nt) database (last accessed on 15th March 2025). Conserved and variable regions among closely related species and strains were identified to ensure comprehensive coverage of sequence variation and to assess their suitability for primer design. Previously established assays were re-evaluated to assess their coverage of the marker databases and recently published sequences from NCBI.

Novel primer pairs were derived using the online primer designing tool Primer3Plus, release v3.2.0 [57]. To enable simultaneous analysis and comparison of all sampled regions, forward primers of the selected markers (Table 3) were labelled at the 5′ end with individual 16-mer nucleotide barcode sequences specific to each region (Pacific Biosciences, Menlo Park, CA, USA). All primers were also checked for specificity via the primer BLAST [58] of the NCBI against the nucleotide collection (nt) database (last accessed on 15th March 2025). All oligonucleotides were synthesised by a local vendor (Metabion GmbH, Planegg/Steinkirchen, Germany).

### 4.5. ‘Ca. A. phytopathogenicus’ Assessment in Sugar Beet and Planthopper Samples

A novel primer pair named SF1/SR1 (Table 8) was designed to amplify the *rplO*-*secY*-*rpmJ* locus, producing a 1511 bp PCR product (based on in silico prediction with the reference genome of ‘*Ca*. A. phytopathogenicus’ strains UHOH and PENLEP) spanning the complete *secY* gene encoding the preprotein translocase subunit SecY, which is flanked by the genes *rplO* and *rpmJ*, that encode the 50S ribosomal proteins L15 and L36. Further, the primer pair GF6/GR6 (Table 8) was developed to amplify the partial sequence of the gene *groEL* coding for the heat-shock protein 60 kDa family chaperone GroEL with a barcoded PCR product of 1618 bp. PCR reactions with the primers were set up in a 15 µL reaction volume using the 1X OneTaq Hot Start Master Mix with Standard Buffer (New England Biolabs GmbH, Frankfurt am Main, Germany), with a primer concentration of 0.2 µM for each primer and an approximate input of 10 ng for each reaction of metagenomic DNA template obtained from sugar beet or planthoppers. Amplification with SF1/SR1 was initiated with a denaturation step at 94 °C for 30 s, followed by 35 amplification cycles comprising denaturation at 94 °C for 15 s, annealing at 60 °C for 30 s, extension at 68 °C for 1 min, and terminated by a final extension at 68 °C for 5 min. Optimal PCR conditions for GF6/GR6 were initial denaturation at 94 °C for 30 s, followed by 35 cycles of denaturation at 94 °C for 30 s, annealing at 64 °C for 30 s, and extension at 68 °C for 2 min 30 s, with a last extension step at 68 °C for 5 min. For evaluation, each PCR run included a positive control consisting of DNA from a confirmed-infected sugar beet or *P*. *leporinus* sample and a non-template control including nuclease-free water instead of DNA. Then, 5 µL of the obtained PCR products were separated on a 1% agarose gel, stained with Safe Gel red (GENAXXON bioscience GmbH, Ulm, Germany), and visualised with a UV-transilluminator (Figures S1 and S2). DNA templates of *P*. *leporinus* were confirmed using specific primers [29] with the OneTaq Hot Start Master Mix with Standard Buffer in 15 µL reactions as described above. Amplification was performed with initiated with a denaturation step at 94 °C for 30 s, followed by 35 amplification cycles comprising denaturation at 94 °C for 30 s, annealing at 56 °C for 30 s, and extension at 68 °C for 30 s, and terminated by a final extension at 68 °C for 5 min. Additionally, as negative controls, DNA templates from *R*. *artemisiae* and *H*. *obsoletus* were utilised. Visualisation of the obtained PCR products was performed as described above.

### 4.6. Assessment of Phytoplasmas in Sugar Beet

#### 4.6.1. Markers for Universal Phytoplasma Detection

For general phytoplasma assessment in sugar beet, the universal primer pair P1/P7 amplifying the 16S rRNA-ITS-23S rRNA region of the ribosomal RNA operon was utilised (Table 8) [59,60]. Generation of barcoded amplicons with a length of 1805 bp (based on in silico prediction with the reference genome ‘*Ca*. P. solani’-related strains GOE and PENLEP) using P1/P7 was performed in 16 µL reactions consisting of 1X Thermo Scientific PCR-Mastermix (Life Technologies GmbH, Darmstadt, Germany) and 0.28 µM for each primer and an approximate input of 10 ng for each reaction of metagenomic DNA template obtained from sugar beet, and filled up with PCR-grade water to the final reaction volume. PCR protocol was performed according to the published protocol [60]. Evaluation of the PCR reaction was performed as described above (Figure S3).

#### 4.6.2. Markers for Group-Specific Detection of Stolbur Phytoplasmas

For group-specific detection of the stolbur phytoplasmas, two novel primer pairs, TuF1/TuR1 and GROLF/NADR, were designed that cover the subgroups 16SrXII-A and 16SrXII-P (Table 8). TuF1/TuR1 was designed for partial amplification of the gene *tuf* (coding for the translation elongation factor Tu), producing an amplicon of 1111 bp in length. The GROLF/NADR primer pair targets the *groEL*-*stamp*-*nadE* locus, generating a 2927 bp long amplicon spanning the complete gene sequence of the stolbur antigenic membrane protein, as well as the partial gene sequences of the 60 kDa family chaperone GroEL and the NAD synthetase. PCR reactions with both novel primer pairs were optimised for 15 µL reactions consisting of 1X KAPA HiFi Hot Start Ready Mix (Roche Diagnostics GmbH, Mannheim, Germany), a primer concentration of 0.2 µM for each primer, and approximately 10 ng input of DNA Template. Following PCR optimisation, final PCR conditions for TuF1/TuR1 were an initial denaturation at 95 °C for 3 min, followed by 35 cycles of denaturation at 98 °C for 30 s, annealing at 64 °C for 15 s, and extension at 72 °C for 2 min, with a final extension at 72 °C for 2 min. For GROLF/NADR, PCR was carried out with an initial denaturation at 95 °C for 3 min, followed by 35 cycles of denaturation at 98 °C for 30 s, annealing at 60 °C for 15 s, and extension at 72 °C for 4 min, concluding with a final extension at 72 °C for 4 min. Evaluation of the PCR reaction was also performed as described before (Figures S4 and S5).

### 4.7. Regional Barcoded Amplicon Pools and Sequencing

To assess genetic diversity, single-end barcoded PCR products were generated using the established PCR assays with barcoded forward primers (Table S3) and a DNA template input of approximately 10 ng per reaction. To create sequencing libraries, all positive samples were utilised for the generation of regional amplicon pools per target. Due to the high number of individual amplicons, initial pooling and normalisation occurred semi-quantitatively based on gel band intensity to regional pools. Subsequently, regional amplicon pools for each target were set up into marker-specific target pools with proportional regional representation according to the number of positive samples for each region (Tables S1 and S2). Marker-specific target pools were quantified fluorometrically with a Qubit fluorometer (Life Technologies GmbH, Darmstadt, Germany) and further pooled to equimolar balanced sequencing pools. Equimolar balanced sequencing pools were purified using the Monarch PCR & DNA Cleanup Kit (5 µg) (New England Biolabs GmbH, Frankfurt am Main, Germany) and checked again for quantity. A final quality check regarding amplicon representation and quantity was performed prior to sequencing with a 2100 Bioanalyzer (Agilent Technologies, Santa Clara, CA, USA). SMRTbell sequencing libraries were prepared using the SMRTbell prep kit v3.0 (Pacific Biosciences, Menlo Park, CA, USA), according to the protocol provided by the vendor. Two prepared libraries were subsequently sequenced in two runs on a PacBio Revio system using a 25M zero-mode waveguide (ZMW) SMRT Cell, together with SPRQ polymerase and SPRQ sequencing chemistry at the Max Planck Genome Centre Cologne (Cologne, Germany). Raw HiFi circular consensus (ccs) read data were processed with the SMRT Link software v25.1.0.257715 (Pacific Biosciences, Menlo Park, CA, USA) after sequencing. Only HiFi ccs reads showing a minimum of three passes in a ZMW and a minimum quality value of 20 were retained for the analysis post sequencing. Further adapters were removed and demultiplexed to the regional subsets according to the used barcodes. Read statistics were assessed with seqkit v2.6.0 [61].

### 4.8. Prediction of Amplicon Sequence Variants

Obtained read data from SMRT sequencing were used to predict amplicon sequence variants (ASVs) with DADA2 v1.3.4 [62]. For ASV prediction, the read data were initially filtered based on the expected amplicon length. A tolerance of approximately ±100 bp was allowed to account for natural variation, sequencing artefacts, and primer imprecision. Furthermore, only reads with a quality value equal to 30 or higher and without N’s were retained in the dataset. Filtered reads were subjected to ASV inference using default settings, with chimeric sequences removed post-prediction. ASVs were inferred separately for each marker and assigned compact study-specific identifiers using marker-specific one-letter prefixes, namely Y (*rplO*-*secY*-*rpmJ*) and G (*groEL*) for ‘*Ca*. A. phytopathogenicus’, and R (16S rRNA-ITS-23S rRNA), T (*tuf*), and S (*groEL-stamp-nadE*) for phytoplasmas. Each prefix was combined with a numerical identifier to distinguish individual ASVs. Predicted ASVs were finally checked for affiliation via the BLAST by application of the MEGABLAST algorithm against the NCBI nucleotide collection database (accessed 15 December 2025) to check target specificity. As a final step, ASVs were manually inspected using the Artemis genome browser release 18.2.0 [63] to identify sequences containing indels that disrupted expected coding sequences, which were excluded from downstream analyses.

### 4.9. Phylogenetic Analysis

#### 4.9.1. Inference of Genetic Clusters

Phylogenetic clustering was based on a selection of reference genome data of the analysed pathogens and closely related species that was retrieved from NCBI. For ‘*Ca*. A. phytopathogenicus’, ASVs were compared with markers obtained from the complete genome sequence of strain PENLEP [33] as well as strains UHOH [31], Ap-FR (JBDPZA000000000.1), and Ap-CH (JBDPZB000000000.1) that represent incomplete draft genomes. Further marker sequences of the nine reference genomes from NCBI of insect-associated endosymbionts of the genus *Arsenophonus* (*A*.) with *A*. endosymbiont of *Crataerina pallida* (OZ026540.1), ‘*Ca*. A. nilaparvatae’ (CP158507.1), *A*. endosymbiont of *Aphis craccivora* (CP038155.1), *A*. endosymbiont of *Aleurodicus disperses* (LR025108.1), *A*. *nasoniae* (CP038613.1), *A*. *apicola* (CP084222.1), ‘*Ca*. A. lipoptenae*’* (CP013920.1), *A*. endosymbiont of *Lipoptena cervi* (OZ195522.1), and *A*. symbiont of *Ornithomya chloropus* (OZ032148.1) were chosen. Additionally, marker sequences of ‘*Candidatus* Phloeobacter fragariae’ strain Pf-FR (JBDPYZ000000000.1) [35,36] were selected as the outgroup to achieve robust topology and statistical confidence.

Phytoplasma ASVs were compared to the markers of the available complete genome sequences of the stolbur group (16SrXII) (accessed 15 December 2025). This included ‘*Ca*. P. solani’ strains c1 (CP103788.1), c4 (CP103787.1), c5 (CP103786.1), and o3 (CP103785.1) as reference for the 16SrXII-A subgroup; markers of the complete genome sequences of the 16SrXII-P strains GOE (CP155828.1) and PENLEP [33]; and markers of ‘*Ca*. P. australiense’ strains PAa (AM422018.) and NZsb11 (CP002548.1) of the subgroups 16SrXII-B and 16SrXII-C. Moreover, ‘*Ca*. P. asteris’, with the strains M33 (CP128397.1) and M8 (CP128414.1) of the subgroups 16SrI-A and 16SrI-B, were also added as outgroups to improve topology and statistical support.

Phylogenetic clustering was based on multiple nucleotide sequence alignments calculated with the MUSCLE algorithm in the Molecular Evolutionary Genetics Analysis (MEGA) software v12.0.10 [64], with default settings. Delineation of genetic lines was performed by constructing maximum-likelihood (ML) trees using IQ-TREE v2.4.0 [65]. Models were selected according to the lowest Bayesian information criterion (BIC) value for optimal balance between model fit and complexity. All trees were calculated with 1000 bootstrap iterations, with a threshold of 70 for statistical significance. Final trees were visualised and edited in MEGA. Clusters were labelled with marker-specific three-letter prefixes: RRO (16S rRNA-ITS-23S rRNA), TUF (*tuf*), GSN (*groEL-stamp-nadE*) for phytoplasmas and SEC/GRO (*rplO*-*secY*-*rpmJ*/*groEL*) for ‘*Ca*. A. phytopathogenicus’, each followed by a number. These designations are operational and ensure consistent referencing throughout this study.

#### 4.9.2. Sequence Difference Analysis

Multiple-sequence alignments produced in the preceding step were used to quantify sequence variation among ASVs and reference sequences at the nucleotide and amino acid levels. Sequence differences and sequence identity matrices were subsequently inferred with BioEdit v7.2.5 [66] to provide a measure of pairwise divergence and marker-specific phylogenetic resolution. For the non-protein coding target 16Sr RNA-ITS-23S rRNA, a minimum of 0.5% variation at the nucleotide level was used to justify a novel cluster additional to the support from the clustering of the ML tree topology, while for the protein-coding sequences, a separate cluster was justified with a minimum of three informative sites in the amino acid sequence in accordance with the topology with the given reference genomes.

#### 4.9.3. Comparison with Reference Data from Sugar Beet

To evaluate the phylogenetic placement of the identified genetic clusters, informative genetic markers were subjected to additional comparative analyses using sugar beet-derived reference sequences retrieved from NCBI. For the 16S rRNA-ITS-23S rRNA marker, inferred ASV clusters were compared against publicly available sugar beet-derived reference sequences. For the *tuf* marker, representative sequences previously published for sugar beet and assigned to the genetic lineages *tuf*-a, *tuf*-b, and *tuf*-d were included. For the *groEL-stamp-nadE* locus, the complete sequence of *stamp* was used for comparison with representative sequences covering all currently available genotypes and sequence variants reported from sugar beet [21,24,25]. Further, to ensure robust clustering, selected reference sequences from other stolbur from the stolbur subgroups, namely, 16SrXII-D of ‘*Ca*. P. japonicum’, 16SrXII-E of ‘*Ca*. P. fragariae’, and 16SrXII-H of ‘*Ca*. P. convolvuli’, as well as references from the other phytoplasmas found in sugar beet from the subgroups 16SrI-B of ‘*Ca*. P. asteris’, 16SrIII-D of Sugar Beet Witches’ Broom phytoplasma and 16SrIII of Sugar Beet Wilt Phytoplasma were added for each marker if available (Table S18). Phylogenetic clustering was re-evaluated using MEGA and IQ-TREE, following the same procedures described above (see Chapter 4.9.2). All analysed markers were trimmed to a comparable sequence length to enable robust clustering.

## 5. Conclusions

In this study, we implemented a long-read amplicon sequencing approach to enable a marker-based high-throughput characterisation of the pathogens ‘*Ca*. A. phytopathogenicus’ and phytoplasmas occurring in SBR-/RTD-affected sugar beet production areas. While ‘*Ca*. A. phytopathogenicus’ displayed marker-based genetic uniformity, phytoplasmas exhibited subgroup-specific diversity patterns, with limited variation in 16SrXII-P and higher lineage-level diversity in 16SrXII-A, consistent with vector-associated epidemiological patterns. Our results support the dominance of ‘*Ca*. A. phytopathogenicus’ and 16SrXII-P phytoplasmas in Germany and extend their known distribution to neighbouring countries. Overall, this work enlarges the molecular foundation for the characterisation and detection of these rapidly spreading sugar beet pathogens and highlights the need for molecular differentiation to support epidemiological understanding and effective disease management.

## Figures and Tables

**Table 1:** Regional pathogen detections among root tip samples from individual sugar beet plants.

Origin	Region	Samples n	Positives n	ARSEPH ^1^ n (%)	PHYP ^2^ n (%)	ARSEPH ^1^ + PHYP ^2^ n (%)
Germany	Heilbronn-Franconia	535	523	6 (1.15%)	188 (35.95%)	329 (62.90%)
	Stuttgart	440	378	60 (15.87%)	190 (50.27%)	128 (33.86%)
	Donau-Isar	44	40	12 (30.00%)	7 (17.50%)	21 (52.50%)
	Saxony-Anhalt/Brandenburg	547	434	76 (17.51%)	132 (30.42%)	226 (52.07%)
	Rhineland-Palatinate	278	221	168 (76.02%)	6 (2.71%)	47 (21.27%)
	North Rhine-Westphalia	81	0	0 (0.00%)	0 (0.00%)	0 (0.00%)
	Total (Germany)	1925	1596	322 (20.18%)	523 (32.77%)	751 (47.05%)
Neighbouring countries	France	130	84	83 (98.81%)	0 (0.00%)	1 (1.19%)
	Switzerland	193	179	178 (100.00%)	0 (0.00%)	0 (0.00%)
	Austria	94	86	0 (0.00%)	86 (100.00%)	0 (0.00%)
	Slovakia	20	2	0 (0.00%)	2 (100.00%)	0 (0.00%)
	Poland	42	17	3 (17.65%)	10 (58.82%)	4 (23.53%)
	The Netherlands	31	0	0 (0.00%)	0 (0.00%)	0 (0.00%)
	Belgium	44	0	0 (0.00%)	0 (0.00%)	0 (0.00%)
	Total (Neighbouring Countries)	554	367	264 (71.94%)	98 (26.70%)	5 (1.36%)
	Total (Overall)	2479	1963	586 (29.85%)	621 (31.64%)	756 (38.51%)

^1^ ARSEPH, ‘*Ca*. A. phytopathogenicus’; ^2^ PHYP, ‘*Ca*. Phytoplasma’.

**Table 2:** Regional detections of the ‘*Ca*. A. phytopathogenicus’ in *P*. *leporinus* samples.

Region	Year	Sample No. n	*P*. *leporinus* n (%)	ARSEPH ^1^ n (%)
Heilbronn-Franconia	2023	40	35 (92.50)	17 (70.00)
Heilbronn-Franconia	2025	40	40 (100.00)	39 (97.50)
Stuttgart		40	40 (100.00)	36 (90.00)
Saxony-Anhalt		40	40 (100.00)	36 (90.00)
Rhineland-Palatinate		40	37 (87.50)	34 (85.00)
Donau-Isar		40	40 (100.00)	32 (80.00)
Total		240	232 (96.67)	194 (80.83)

^1^ ARSEPH, ‘*Ca*. A. phytopathogenicus’.

**Table 3:** Overview of produced read data from positive samples.

Target Organism	Marker	Host	Positives n	Reads (Unfiltered) n (%)	Average Length nt	Reads (Passed) n (%)
‘*Ca*. A. phytopathogenicus’	*rplO*-*secY*-*rpmJ*	sugar beet	1218 1284	1,335,675	1470	708,244
	*groEL*			1,279,921	1555	603,426
		*P*. *leporinus*	159 177	393,201 442,077	1454 1561	168,863 162,860
Total (‘*Ca*. A. phytopathogenicus’)				3,450,874 (30.79%)		1,643,393 (31.20%)
‘*Ca*. Phytoplasma’	16S rRNA-ITS-23S rRNA		1185	3,875,213	1745	1,578,874
	*tuf*		776	1,522,102	1046	972,009
	*groEL-stamp-nadE*		1133	2,361,326	2865	1,074,522
Total (‘*Ca*. Phytoplasma’)				7,758,641 (69.21%)		3,625,405 (68.80%)
Total (Overall)			5932	11,209,515		5,268,798

**Table 4:** Overview of inferred ASV cluster abundances per marker.

Target Organism	Marker	Read No	Subgroup	ASVs n	Cluster	Reads in Cluster n	Relative Abundance %
‘*Ca*. A. phytopathogenicus’	*rplO*-*secY*-*rpmJ*	877,107	-	1	SEC1	877,107	100
	*groEL*	766,286	-	1	GRO1	766,286	100
‘*Ca*. Phytoplasma’	16S rRNA-ITS-23S rRNA	1,578,887	16SrXII-A	4 1 2	RRO-1 RRO-2 RRO-3	126,435 95 210	8.008 0.006 0.013
			16SrXII-P	6 1	RRO-4 RRO-5	1,451,689 143	91.943 0.009
			16SrI-A	1	RRO-6	334	0.021
‘*Ca*. P. solani’	*tuf*	972,009	16SrXII-A	3	TUF-1	1153	0.119
			16SrXII-P	15	TUF-2	970,856	99.881
	*groEL-stamp-nadE*	1,074,571	16SrXII-A	2 1 1 1	GSN-1 GSN-2 GSN-3 GSN-4	9401 14 288 18	0.875 0.001 0.027 0.002
			16SrXII-P	9	GSN-5	1,064,853	99.096

Read distributions per ASV and region, as well as the ASV abundances within the complete marker sets, are reported in Tables S4–S8 in the Supplementary Materials.

**Table 5:** Overview of marker sequence variation within obtained ASV clusters.

Target Organism	Marker	Subgroup	Cluster	Inter-Subgroup Identity %	Inter-Cluster Identity %	Intra-Cluster Identity %	Most Variable Region
‘*Ca*. A. phytopathogenicus’	*rplO*-*secY*-*rpmJ*	-	SEC-1	-	-	100	-
	*groEL*	-	GRO-1	-	-	100	-
‘*Ca*. Phytoplasma’	16S rRNA-ITS-23S rRNA	16SrXII-A	RRO-1	97.2–99.3	98.1–99.8	98.1–100	16S rRNA
			RRO-2			-	
			RRO-3			99.9	
		16SrXII-P	RRO-4		90–99.3	99.7–100	23S rRNA
			RRO-5			-	
		16SrI-A	RRO-6	-	99.2–92.6	-	
‘*Ca*. P. solani’	*tuf*	16SrXII-A	TUF-1	89.5–89.1	-	99.7–100	-
		16SrXII-P	TUF-2		-	99.8–100	
	*groEL-stamp-nadE*	16SrXII-A	GSN-1	73.1–73.9	98.8–99.4	99.8	*stamp*
			GSN-2			-	
			GSN-3			100	
			GSN-4			-	
		16SrXII-P	GSN-5		-	99.9	*groEL*

**Table 6:** Sugar beet samples with locations from 2023 and 2024 used for diversity assessment.

Year	Sampling	Origin	Region	Location	Geo Coordinates	Sample Numbers
2023	Positives selection of random clamp samples	Germany	Stuttgart	Tiefenbronn Pulverdingen	48°48′20.5″ N 8°50′07.3″ E 48°54′16.1″ N 9°01′19.4″ E	202 124
			Heilbronn-Franconia	Leingarten Schwaigern Obernickelsheim Gaukönigshofen	49°09′29.6″ N 9°04′47.0″ E 49°10′16.6″ N 9°03′49.8″ E 49°34’48.2″ N 10°08’24.2″ E 49°37’24.4″ N 9°57’07.9″ E	68 103 100 99
			Donau-Isar	Zöschingen Sonderheim	48°40’04.5″ N 10°19’42.6″ E 48°37’49.6″ N 10°35’49.4″ E	3 3
			Saxony- Anhalt	Wörbzig Glinde-Pömmelte Samswegen-Hillersleben	51°42′46.3″ N 11°54′55.2″ E 52°00′08.0″ N 11°50′10.9″ E 52°16′41.1″ N 11°28′59.1″ E	21 98 62
			Rhineland- Palatinate	Otterstadt Bischheim Morschheim	49°22′00.1″ N 8°27′33.4″ E 49°21′53.3″ N 8°27′47.8″ E 49°40′31.8″ N 8°01′05.4″ E 49°41′21.3″ N 8°01′30.9″ E	85 97 10 10
		Neighbouring countries	France	Sundhouse Schoenau	48°14′28.0″ N 7°38′34.0″ E 48°14′31.6″ N 7°39′14.3″ E	79 31
			Switzerland	Engolon Müntschemier	47°02′38.7″ N 6°55′43.2″ E 47°00′10.9″ N 7°08′31.7″ E	92 101
			Austria	Großkrut Patzmannsdorf	48°39′45.2″ N 16°44′56.2″ E 48°38′07.7″ N 16°16′21.2″ E	49 5
			Poland	Tychnowy Pawłowiczki	53°47′16.9″ N 18°58′37.2″ E 50°16′10.4″ N 18°03′40.9″ E	1 1
			The Netherlands	Grashoek Bant Roermond	51°21′24.5″ N 5°56′29.0″ E 52°46′01.1″ N 5°43′46.5″ E 51°11′37.6″ N 6°03′28.2″ E	7 3 1
			Belgium	Brakel	50°46′37.4″ N 3°44′43.1″ E	4
2024	Symptomatic field samples	Germany	Heilbronn-Franconia	Bad Wimpfen Hüffenhardt Rodheim Eppingen	49°12′33.7″ N 9°09′28.6″ E 49°17′39.2″ N 9°03′09.2″ E 49°34′49.3″ N 10°09′05.9″ E 49°08′52.9″ N 8°53′40.7″ E	41 40 44 40
			Stuttgart	Oberriexingen Ditzingen Pulverdingen	48°56′38.6″ N 9°02′28.8″ E 48°49′48.1″ N 9°04′53.3″ E 48°53′53.3″ N 9°01′43.0″ E	40 40 34
			Saxony-Anhalt /Brandenburg	Gehrden Hobeck Gommern-Nedlitz Vockerode Bageritz Buhlendorf Ottersleben Neureetz (Oderaue)	52°01′09.0″ N 11°59′30.3″ E 52°04′00.3″ N 12°03′17.7″ E 52°07′14.2″ N 11°48′45.2″ E 51°50′22.7″ N 12°22′24.4″ E 51°28′55.6″ N 12°09′28.6″ E * 52°02′08.5″ N 12°01′35.8″ E 52°04′40.1″ N 11°33′50.5″ E 52°47′01.1″ N 14°08′26.7″ E	45 41 41 40 80 40 40 40
			Rhineland-Palatinate	Worms-Eich Bischheim	49°45′29.1″ N 8°25′31.2″ E 49°40′07.1″ N 8°02′53.5″ E	41 35
			Donau-Isar	Oberdolling	48°50′33.7″ N 11°36′38.9″ E	38
			North Rhine-Westphalia	Bornheim Vettweiß	50°47′08.2″ N 6°55′36.9″ E 50°45′15.3″ N 6°40′48.6″ E	41 40
		Neighbouring countries	France	Richtolsheim	48°13′13.3″ N 7°36′48.3″ E	20
			Austria	Ginzersdorf Jedenspeigen	48°37′39.1″ N 16°42′06.2″ E 48°31′15.8″ N 16°50′19.5″ E	20 20
			Slovakia	Malé Zálužie	48°25′45.6″ N 17°59′15.5″ E	20
			Poland	Siedlisko-Dębianka Piotrowice	51°44′46.2″ N 15°51′58.7″ E 51°51′56.7″ N 16°24′34.2″ E	20 20
			The Netherlands	Orvelte	52°51′07.1″ N 6°39′51.4″ E	20
			Belgium	Verlaine Donceel	50°36′41.8″ N 5°19′55.6″ E 50°39′48.3″ N 5°19′16.4″ E	20 20

Coordinates marked with an asterisk represent approximate locations.

**Table 7:** Overview of cicada sampling numbers and locations in 2023 and 2025.

Year	Region	Location	Geo Coordinates	Sample Number
2023	Heilbronn-Franconia	Kirchhausen	49°10′54.5″ N 9°05′37.7″ E	40
2025	Heilbronn-Franconia	Treschklingen Gelchsheim	49°13′17.1″ N 9°03′32.3″ E 49°33′40.0″ N 10°00′41.2″ E	20 20
	Stuttgart	Ditzingen Hirschlanden	48°49′38.1″ N 9°00′47.4″ E 48°49′29.9″ N 9°01′06.0″ E	20 20
	Saxony-Anhalt	Kemberg Arzberg	51°47′47.0″ N 12°39′22.5″ E 51°30′32.9″ N 13°09′23.2″ E	20 20
	Rhineland-Palatinate	Worms-Eich	49°44′53.6″ N 8°25′42.2″ E	40
	Donau-Isar	Ingoldstadt	48°42′53.1″ N 11°23′12.9″ E 48°42′40.7″ N 11°22′16.4″ E	20 20

**Table 8:** Overview of primer pairs used for diversity assessment.

Target Organism	Primer	Sequence (5′ to 3′)	Marker	Amplicon Length (bp)	References
‘*Ca*. A. phytopathogenicus’	SF1 SR1	CACGGACTGCAATTGAAGCG CACACGAACAATACCTTCACGT	*rplO-secY-rpmJ*	1511	This study
	GF6 GR6	ACCACCCATACCACCCATACCAGCA GGGATTGACGCCCGTGCAAAAAT	*groEL*	1618	This study
‘*Ca*. Phytoplasma’	P1 P7	AAGAGTTTGATCCTGGCTCAGGATT CGTCCTTCATCGGCTCTT	16S rRNA-ITS-23S rRNA	1805	[59,60]
‘*Ca*. P. solani’	TuF1 TuR1	ACGGAAAAACCATTTAACAGCTGCCA ATCCGGCACCAACTGTTTTACCACC	*tuf*	1111	This study
	GROLF NADR	GGCGCAAAGTATGATTCATCGTGG CCACCTATTTGCTTGCGAGAATG	*groEL*-*stamp*-*nadE*	2927	This study

Amplicon lengths are based on the complete genome sequence of ‘*Ca*. P. solani’-related strains GOE [38] and PENLEP [33] and the ‘*Ca*. A. phytopathogenicus’ strains UHOH [31] and PENLEP [33], including the length of 16 nt of the barcode sequences attached to the forward primer.

## Data Availability

Data have been deposited in the NCBI database under BioProject (PRJNA1445063) and BioSamples (SAMN56777072, SAMN56777073, SAMN56777074). ASVs for all analysed marker loci have been deposited in GenBank and are available under the following accessions: 16S rRNA-ITS-23S rRNA (PZ229018-PZ229031), *tuf* (PZ224554–PZ224571), *groEL*-*stamp*-*nadE* (PZ224572–PZ224585) for phytoplasmas, and *rplO*-*secY*-*rpmJ* (PZ224587) and *groEL* (PZ224586) for ‘*Ca.* A. phytopathogenicus’.

## Supplementary Materials

The following supporting information can be downloaded at: https://www.mdpi.com/article/10.3390/plants15111618/s1, Figures S1–S5: Representative agarose gel pictures of the applied PCR assays with barcoded primers; Tables S1 and S2: Overview of regional detection for phytoplasmas and ‘*Ca*. A. phytopathogenicus’ in the years 2023 and 2024.; Table S3: Overview of sequencing results per barcode primer; Tables S4–S8: Reads per ASV and regional distribution; Tables S9–S13: Pairwise nucleotide identities of ASVs and reference sequences; Tables S14–S17A–C: Pairwise differences in amino acid sequences of the analysed ASVs and reference data, Table S18: 16SrRNA sequences within collapsed clusters from analysis with reference sequences of other stolbur subgroups.
