# Supplementary material for: Diversity Analysis of the Sugar Beet Pathogens ‘Candidatus Arsenophonus phytopathogenicus’ and ‘Ca. Phytoplasma solani’
Source: Plants (Basel). 2026 May 25;15(11):1618. doi: 10.3390/plants15111618 (PMC13259348; doi:10.3390/plants15111618)
Supplement: Supplementary file 1 [file plants-15-01618-s001.zip › plants-4279497-Supplementary Material Figures.pdf]

Supplementary Material

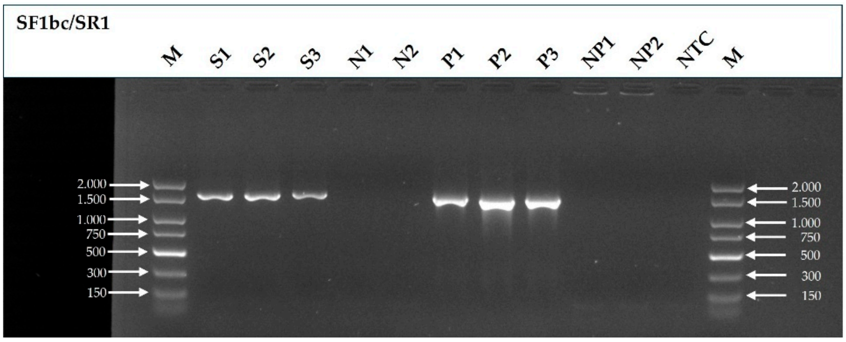

Figure S1. Representative 1% agarose gel used for validation SF1bc/SR1 PCR assay. M, molecular size marker; S1–3, representative DNA templates from sugar beets positive for '*Ca*. A. phytopathogenicus'; N1-2, DNA-Templates from sugar beet tested negative for '*Ca*. A. phytopathogenicus'; sugar beets; P1-3, DNA-Templates from '*Ca*. A. phytopathogenicus' infected *P. leporinus*; NP1-2, DNA-Templates from *P. leporinus* tested negative for '*Ca*. A. phytopathogenicus'; NTC, non-template control.

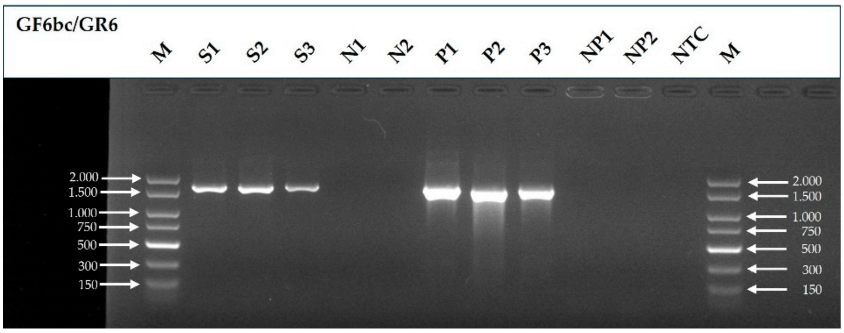

Figure S2. Representative 1% agarose gel used for validation of the GF61bc/GR6 PCR assay. M, molecular size marker; S1–3, representative DNA templates from sugar beets positive for '*Ca*. A. phytopathogenicus'; N1-2, DNA-Templates from sugar beet tested negative for '*Ca*. A. phytopathogenicus'; sugar beets; P1-3, DNA-Templates from '*Ca*. A. phytopathogenicus' infected *P. leporinus*; NP1-2, DNA-Templates from *P. leporinus* tested negative for '*Ca*. A. phytopathogenicus'; NTC, non-template control.

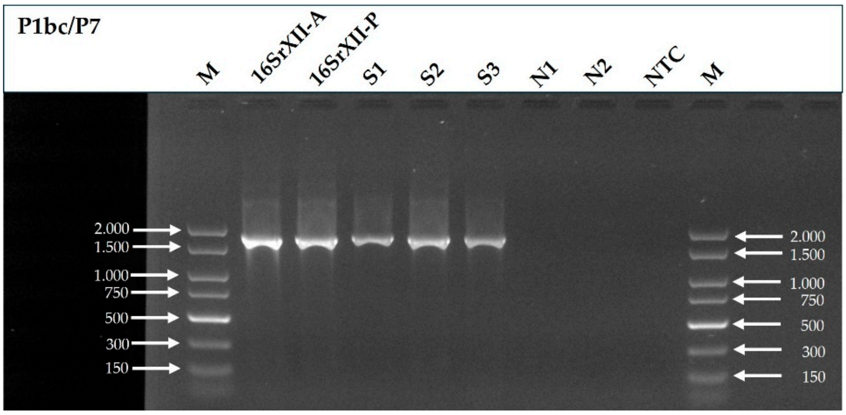

Figure S3. Representative 1% agarose gel used for validation of the P1bc/P7 PCR assay. M, molecular size marker; 16SrXII-A, DNA templates from a sugar beet infected with '*Ca*. P. solani' subgroup 16SrXII-A; 16SrXII-P, DNA templates from a sugar beet infected with '*Ca*. P. solani'-related phytoplasma subgroup 16SrXII-P; S1–3, representative DNA templates from sugar beets positive for '*Ca*. Phytoplasma'; N1-2, DNA-Templates from sugar beet tested negative for '*Ca*. Phytoplasma'; sugar beets; NTC, non-template control.

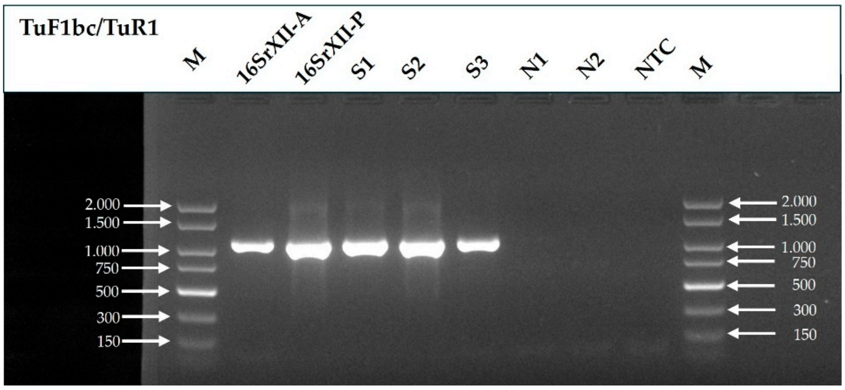

Figure S4. Representative 1% agarose gel used for validation of the P1bc/P7 PCR assay. M, molecular size marker; 16SrXII-A, DNA templates from a sugar beet infected with '*Ca*. P. solani' subgroup 16SrXII-A; 16SrXII-P, DNA templates from a sugar beet infected with '*Ca*. P. solani'-related phytoplasma subgroup 16SrXII-P; S1–3, representative DNA templates from sugar beets positive for '*Ca*. Phytoplasma'; N1-2, DNA-Templates from sugar beet tested negative for '*Ca*. Phytoplasma'; sugar beets; NTC, non-template control.

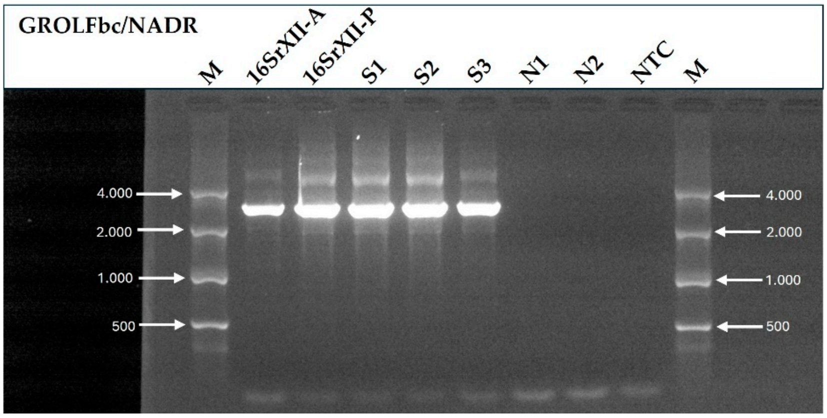

Figure S5. Representative 1% agarose gel used for validation of the GROLFbc/NADR PCR assay. M, molecular size marker; 16SrXII-A, DNA templates from a sugar beet infected with '*Ca*. P. solani' subgroup 16SrXII-A; 16SrXII-P, DNA templates from a sugar beet infected with '*Ca*. P. solani'-related phytoplasma subgroup 16SrXII-P; S1–3, representative DNA templates from sugar beets positive for '*Ca*. Phytoplasma'; N1-2, DNA-Templates from sugar beet tested negative for '*Ca*. Phytoplasma'; sugar beets; NTC, non-template control.
